# Clinical outcomes and spinal growth after posterior hemivertebra resection and short segment fusion in children

**DOI:** 10.1038/s41598-024-53290-y

**Published:** 2024-02-02

**Authors:** Yuxuan Du, Hongqi Zhang, Yuxiang Wang

**Affiliations:** 1 Department of Spine Surgery and Orthopaedics, Xiangya Spinal Surgery Center, Xiangya Hospital of Central South University, Xiang Ya Road 87, Changsha, China; 2 From National Clinical Research Center for Geriatric Disorder, Xiangya Hospital of Central South University, Changsha, China

**Keywords:** Outcomes research, Skeleton

## Abstract

To evaluate the corrective effect of posterior hemivertebra resection and short-segment fusion surgery on pediatric patients and to assess the impact of short-segment fixation surgery on vertebral development during follow-up, a retrospective analysis was performed on 28 pediatric patients who underwent posterior hemivertebra resection surgery. The corrective effect was evaluated by comparing indicators such as segmental scoliosis Cobb angle, upper and lower compensatory curves and trunk balance at different time points. Meanwhile, the vertebral and spinal canal diameters of instrumented vertebrae and adjacent noninstrumented vertebrae were measured and compared to assess vertebral and spinal canal development. The correction rate of segmental scoliosis was 72.2%. The estimated mean vertebral volume of the instrumented vertebra was slightly lower than that of the unfused segment at the final follow-up, but the difference was not statistically significant. The growth rate of the spinal canal during follow-up was much smaller than that of the vertebral body. In summary, internal fixation at a young age shows no significant inhibitory effects on spinal development within the fusion segment. Posterior hemivertebra resection and short-segment fusion surgery are safe and effective.

## Introduction

Hemivertebra is a common cause of congenital scoliosis, which is a developmental defect formed during embryonic development^1,2^. Most hemivertebrae have growth potential and can lead to scoliosis during growth, the degree of which depends on the type, location, and size of the hemivertebra. The resulting asymmetric spinal growth causes not only physical deformities but also psychological distress^3^.

Non-surgical interventions exhibit restricted efficacy for hemivertebrae, with most cases necessitating surgical treatment^4^. Hemivertebrae retain the potential for ongoing progression during growth, potentially leading to alterations in adjacent vertebral structures. Consequently, early-stage surgical intervention becomes imperative to prevent local progression and occurrence of secondary deformities^5^. Among the available surgical modalities, posterior hemivertebra resection combined with short-segment fusion fixation surgery not only provides satisfactory corrective results but also ensures fusion stability while effectively reducing surgical trauma and complications.

The resection of the hemivertebra is a crucial intervention, significantly mitigating the ongoing progression of deformity. A posterior pedicle screw fixation system not only achieves satisfactory corrective outcomes but also ensures robust fixation^6^. However, there is little literature on the impact of the internal fixation system on the development of the vertebral body and spinal canal in children during long-term follow-up.

This study is a retrospective study of patients who underwent hemivertebra resection and short-segment fusion surgery, with a follow-up duration exceeding 5 years. This study evaluates the long-term efficacy of this procedure by assessing parameters such as segmental scoliosis, compensatory curves, segmental kyphosis, and trunk balance. Additionally, the impact of short-segment internal fixation surgery on vertebral and spinal canal development is examined through measurements of vertebral diameters and spinal canal diameters.

## Results

### General data

In this study, a total of 28 patients who had undergone hemivertebra resection and short segment fusion surgery in our hospital between January 2012 and June 2017 were enrolled, including 13 males and 15 females, aged 3–7 years, with an average age of 5.07 ± 1.25 years. Among them, there were 19 cases of fully segmented hemivertebrae and 9 cases of incompletely segmented hemivertebrae. The hemivertebrae were located in the thoracic spine in 16 cases and in the lumbar spine in 12 cases (Table 1).

**Table 1:** General data.

Case number	Sex	Age (years)	Segmentation of Hemivertebra	Hemivertebra level	Follow-up time (months)	Age at last follow-up (years)
1	M	7	Fully segmented	T10	84	14
2	M	6	Fully segmented	T11	84	13
3	F	6	Incompletely segmented	L5	60	11
4	F	5	Incompletely segmented	L3	84	12
5	M	7	Fully segmented	L2	72	13
6	M	6	Fully segmented	T10	48	10
7	F	3	Incompletely segmented	T11	108	12
8	F	7	Fully segmented	L3	72	13
9	F	4	Fully segmented	T11	72	10
10	M	6	Fully segmented	T9	60	11
11	M	4	Incompletely segmented	L1	72	10
12	M	6	Fully segmented	T9	60	11
13	F	4	Fully segmented	L2	72	10
14	M	4	Fully segmented	L3	60	9
15	M	5	Fully segmented	L3	72	11
16	F	6	Fully segmented	T12	72	12
17	M	7	Fully segmented	L1	96	15
18	F	3	Incompletely segmented	T11	84	10
19	M	4	Incompletely segmented	T7	96	12
20	F	5	Fully segmented	L3	120	15
21	F	4	Fully segmented	T9	60	9
22	F	4	Fully segmented	T8	60	9
23	F	6	Incompletely segmented	L2	72	12
24	F	4	Fully segmented	T10	72	10
25	F	5	Fully segmented	T12	72	11
26	M	6	Incompletely segmented	T11	60	11
27	F	3	Fully segmented	L3	96	11
28	M	5	Incompletely segmented	T12	108	14

**Figure 1:**
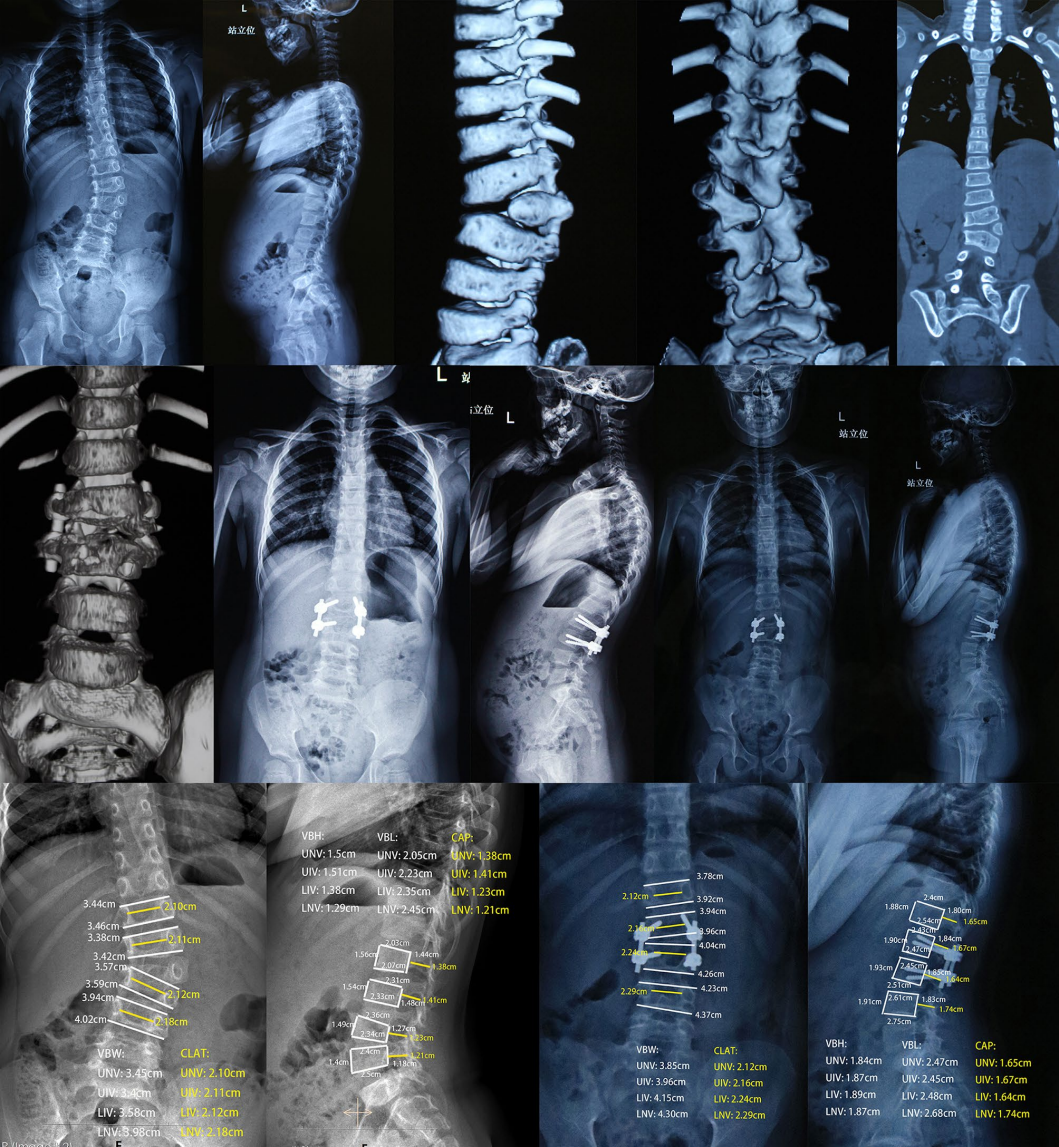
The imaging data of a 4-year-old L2 hemivertebra patient who underwent L2 hemivertebra resection and short-segment fusion surgery with a 6-year follow-up. (**a**) and (**b**) Preoperative X-ray showed that the segmental scoliosis angle of the L2 hemivertebra was 35°, CBD was 0.3 cm, and VSA was 0.5 cm. (**c**-**e**) Preoperative CT scan showed complete segmentation of the hemivertebra. (**f**) Postoperative CT showed complete hemivertebra resection. (**g**) and (**h**) Postoperative X-ray showed that the segmental scoliosis angle was corrected to 4.4°, CBD was 1.45 cm, and SVA was 4.2 cm, which was increased compared to before surgery. (**i**) and (**j**) X-ray at the 6-year follow-up showed that the segmental scoliosis angle was 7.9°, CBD was 0.2 cm, and SVA was 0.4 cm, which was significantly improved compared to before. (**k**) and (**l**) The diameters of vertebral body and spinal canal in different vertebrae before surgery. (**m**) and (**n**) T diameters of vertebral body and spinal canal in different vertebrae before surgery at final follow-up.

The surgical duration was 120–240 min, with an average of 160.71 min, and the blood loss was 150–400 ml, with an average of 219.64 ml. Due to the small amount of blood loss, no transfusion was recorded (Fig. 1).

### Corrective outcome

The segmental scoliosis Cobb angle was corrected from 35.49° preoperatively to 9.87° postoperatively, with a correction rate of 72.2%. The cranial compensatory curve was corrected from 12.79° preoperatively to 3.49° postoperatively, with a correction rate of 67.28%. The caudal compensatory curve was corrected from 18.17° preoperatively to 4.51° postoperatively, with a correction rate of 74.17%. The segmental kyphosis angle was corrected from 15.28° preoperatively to 3.99° postoperatively, with a correction rate of 84.85%. No significant correction loss was found during follow-up (Table 2).

**Table 2:** Corrective outcome of surgery.

	Preoperative	Postoperative	P1	Correction rate (%)	Last follow-up	P2
Coronal indicators						
Segmental scoliosis (°)	35.49 ± 5.73 (28 to 53)	9.87 ± 3.27 (3 to 19.3)	< 0.001	72.2 ± 9.30	9.19 ± 3.35 (3.6 to 18.5)	0.503
Cranial compensatory curve (°)	12.79 ± 5.90 (3.4 to 24.5)	3.49 ± 2.76 (2 to 10.8)	< 0.001	67.28 ± 27.29	3.91 ± 3.04 (1 to 12.3)	0.586
Caudal compensatory curve	18.17 ± 7.64 (3 to 35.1)	4.51 ± 4.01 (1.2 to 11.9)	< 0.001	74.17 ± 23.17	4.37 ± 2.40 (1 to 11.8)	0.753
CBD (cm)	1.18 ± 1.21 (0.2 to 4.5)	0.47 ± 0.50 (0 to 1.75)	0.005	39.29 ± 91.20	0.38 ± 0.49 (0 to 2.1)	0.484
UIV tilt angle (°)	18.66 ± 3.81 (10 to 29)	5.20 ± 2.36 (2.2 to 12.3)	< 0.001	71.40 ± 13.20	4.86 ± 2.38 (2.1 to 13.5)	0.368
LIV tilt angle (°)	16.78 ± 3.42 (11 to 24)	4.40 ± 1.76 (1 to 9)	< 0.001	71.18 ± 10.27	4.41 ± 1.76 (2 to 8)	0.223
Sagittal indicators						
Thoracic kyphosis (°)	28.10 ± 9.36 (12.8 to 51)	25.84 ± 5.75 (8.2 to 34.1)	0.282	5.20 ± 39.47	25.39 ± 4.69 (16.5 to 33)	0.539
Lumbar lordosis (°)	30.13 ± 9.74 (3.5 to 49.8)	30.34 ± 5.47 (17.4 to 43.9)	0.093	7.23 ± 79.22	25.53 ± 5.33 (20 to 37.6)	0.085
Segmental kyphosis (°)	15.28 ± 12.13 (6 to 30.2)	3.99 ± 5.30 (− 5 to 13.5)	< 0.001	84.85 ± 55.75	3.75 ± 4.97 (− 7.1 to 13.6)	0.511
SVA (cm)	0.72 ± 1.22 (0.2 to 3.6)	0.84 ± 1.39 (0 to 1.53)	0.743	54.99 ± 52.75	0.73 ± 1.14 (0 to 2.38)	0.741

Values are presented as mean ± standard deviation (range).

P1: Paired t test *p* value of preoperative and postoperative indicators.

P2: Paired t test *p* value of postoperative and final follow-up indicators.

### Growth of vetebral body

In the comparative analysis between preoperative and final follow-up assessments, varying degrees of growth were observed in the VBW, VBH and VBL across different vertebrae. Notably, among these dimensions, VBH exhibited the highest growth rate compared to the other two parameters (Table 3).

**Table 3:** Vertebral body growth during follow-up.

	Preoperative (cm)	Final follow-up (cm)	*p* value	ΔVBW (cm)	Increase rate (%)
VBW					
UNV	2.91 ± 0.35 (2.3–3.6)	3.16 ± 0.49 (2.4–4.1)	< 0.001	0.25 ± 0.31 (0.1–1)	11.13 ± 11.79
UIV	3.01 ± 0.36 (2.4–3.8)	3.26 ± 0.42 (2.6–4.1)	< 0.001	0.25 ± 0.24 (0.2–0.6)	10.21 ± 8.69
LIV	3.27 ± 0.45 (2.6–4.1)	3.44 ± 0.53 (2.7–4.5)	0.033	0.16 ± 0.41 (0.1–1.1)	6.62 ± 13.63
LNV	3.39 ± 0.38 (2.8–4.5)	3.70 ± 0.57 (2.9–5.6)	< 0.001	0.31 ± 0.37 (0.1–1.4)	11.29 ± 10.44

	Preoperative (cm)	Final follow-up (cm)	*p* value	ΔVBH (cm)	Increase rate (%)
VBH					
UNV	1.38 ± 0.21 (0.9–1.8)	1.84 ± 0.32 (1.2–2.7)	< 0.001	0.45 ± 0.28 (0.1–1.1)	37.98 ± 23.28
UIV	1.44 ± 0.21 (1.1–1.9)	1.85 ± 0.26 (1.3–2.5)	< 0.001	0.41 ± 0.22 (0.2–0.9)	32.77 ± 17.86
LIV	1.52 ± 0.22 (1.0–1.9)	1.97 ± 0.36 (1.5–2.6)	< 0.001	0.45 ± 0.27 (0.2–1.2)	31.71 ± 16.64
LNV	1.57 ± 0.18 (1.3–2.1)	2.11 ± 0.49 (1.4–2.8)	0.002	0.53 ± 0.32 (0.1–1.2)	31.60 ± 19.83

	Preoperative (cm)	Final follow-up (cm)	*p* value	ΔVBL (cm)	Increase rate (%)
VBL					
UNV	2.04 ± 0.27 (1.4–2.4)	2.29 ± 0.31 (1.7–2.7)	< 0.001	0.34 ± 0.24 (0.1–0.9)	22.25 ± 14.53
UIV	2.10 ± 0.24 (1.8–2.5)	2.38 ± 0.29 (2–2.9)	< 0.001	0.28 ± 0.21 (0.1–0.8)	16.69 ± 10.07
LIV	2.08 ± 0.35 (1.4–2.9)	2.34 ± 0.28 (1.8–3)	0.003	0.26 ± 0.28 (0.2–0.9)	18.49 ± 16.59
LNV	2.24 ± 0.32 (1.7–2.7)	2.49 ± 0.36 (1.9–3.5)	0.001	0.25 ± 0.28 (0.2–0.8)	15.07 ± 13.13

Values are presented as mean ± standard deviation (range).

Δ indicates the difference between the measurement data before and at the last follow-up.

Pv represents an estimate of the change in vertebral body volume, determined by the ratio of the product of diameters (VBW, VBH, VBL) measured at the last follow-up to the product of preoperative diameters. ANOVA analysis indicates that there is no statistically significant difference in Pv among different vertebrae (a = 0.515) (Table 5).

### Growth of spinal canal

In the measurements of spinal canal development, the results indicate an increase in both CLAT and CAP in different vertebrae at the last follow-up compared to preoperative values. However, the growth rate of the spinal canal is significantly lower than that of the vertebral bodies (Table 4).

**Table 4:** Spinal canal growth during follow-up.

	Preoperative (cm)	Final follow-up (cm)	*p* value	ΔCLAT (cm)	Increase rate (%)
CLAT					
UNV	1.83 ± 0.26 (1.2–2.4)	1.93 ± 0.28 (1.3–2.6)	0.017	0.20 ± 0.19 (0–0.6)	11.28 ± 10.78
UIV	1.85 ± 0.29 (1.5–2.4)	1.97 ± 0.38 (1.6–2.7)	0.083	0.12 ± 0.18 (0.1–0.7)	6.23 ± 8.53
LIV	2.05 ± 0.34 (1.7–2.7)	2.08 ± 0.42 (1.7–3.3)	0.040	0.03 ± 0.33 (0–1.1)	1.81 ± 14.36
LNV	1.93 ± 0.28 (1.4–2.9)	2.07 ± 0.32 (1.5–3.2)	0.355	0.13 ± 0.26 (0–1.0)	8.15 ± 18.97

	Preoperative (cm)	Final follow-up (cm)	*p* value	ΔCAP (cm)	Increase rate (%)
CAP					
UNV	1.35 ± 0.18 (0.9–1.8)	1.46 ± 0.18 (1.1–1.9)	0.014	0.10 ± 0.18 (0–0.3)	8.44 ± 13.68
UIV	1.37 ± 0.13 (1.1–1.6)	1.43 ± 0.16 (1.2–1.8)	0.026	0.06 ± 0.14 (0–0.4)	5.04 ± 10.76
LIV	1.38 ± 0.16 (1.0–1.7)	1.45 ± 0.19 (1.1–1.8)	0.012	0.06 ± 0.16 (0.1–0.4)	5.12 ± 12.34
LNV	1.41 ± 0.19 (1.0–1.6)	1.48 ± 0.20 (1.1–1.9)	0.011	0.07 ± 0.17 (0–0.5)	5.92 ± 12.78

Values are presented as mean ± standard deviation (range).

Δ indicates the difference between the measurement data before and at the last follow-up.

Pa represents the ratio of the product of CLAT and CAP at the last follow-up to the preoperative values, providing an estimation of the change in the spinal canal area. ANOVA analysis indicates that there is no statistically significant difference in Pv among different vertebrae (a = 0.189) (Table 5).

**Table 5:** Pv and Pa in different vertebrae.

	Pv	Pa
UNV	1.92 ± 0.71 (1.16–2.81)	1.18 ± 0.18 (1.12–1.52)
UIV	1.72 ± 0.38 (1.09–2.68)	1.14 ± 0.17 (1.09–1.61)
LIV	1.67 ± 0.42 (1.08–2.85)	1.08 ± 0.19 (1.08–1.71)
LNV	1.74 ± 0.52 (1.20–2.71)	1.17 ± 0.19 (1.05–1.61)
ANOVA	a = 0.515	a = 0.189

Values are presented as mean ± standard deviation (range).

### Complications

Among the 28 patients, two individuals experienced postoperative superficial incisional infections. Both cases achieved full recovery after a comprehensive treatment involving intensified antibiotic therapy, wound dressing changes, and nutritional support. Throughout the follow-up period, only one case exhibited an “adding-on” phenomenon; however, given its minimal angular deviation and inconspicuous impact on appearance, a secondary surgical intervention was deemed unnecessary. None of the patients experienced trunk and lower limb sensory abnormalities or motor impairments after surgery. No neurological complication was observed in any of the patients during the postoperative period and follow-up.

## Discussion

For progressive hemivertebra, nonsurgical treatment proves ineffective in preventing deformity progression. The literature widely supports the use of surgical treatment in progressive curves^7^. The surgical treatment aims to correct the deformity and balance the trunk. Early surgical intervention can help save the fusion range and preserve spinal mobility and growth potential of unaffected segments^8^. In this study, we focused on the corrective effect of hemivertebra resection and short segment fusion surgery and the influence of internal fixation on spinal growth.

### Corrective effect

Compared to alternative approaches, posterior hemivertebra resection and short segment fusion surgery are currently the most widely used surgical methods^8,9^. In our study, this procedure was employed for all patients. Utilizing wedge osteotomy or complete hemivertebra removal allowed for a more substantial correction angle within a single segment^7^. Benefiting from the favorable flexibility and relatively small scoliosis angles, the correction rate of the segmental scoliosis was 72.2%. Throughout the follow-up period, although some patients experienced slight correction loss, it did not significantly impact the overall outcome. The correction rates for cranial and caudal compensatory curves were 67.28% and 74.17%, respectively. Due to the inherent flexibility of the pediatric spine, substantial correction of compensatory curves could be attained following adequate correction of the structural curve (Table 2).

The orthopedic results indicate that the correction rate of segmental kyphosis is lower than that of segmental scoliosis. In cases enrolled in our study, deformities primarily manifest as lateral curvature in the coronal plane. In some patients, the kyphosis deformity is not as pronounced, resulting in a relatively lower correction rate. Meanwhile, in another subset of patients with a larger kyphotic angle, satisfactory kyphosis correction can be achieved by removing the posterior elements of the hemivertebra and compressing the fixation rods. Throughout the follow-up period, both CBD and SVA showed further reduction, likely attributed to the spontaneous postural correction during growth^10^. Only one case of the Adding-On phenomenon occurred during follow-up among all patients, which may be related to incomplete hemivertebra resection and a larger angle of UIV tilt^11,12^.

### Spinal growth after surgery

Having undergone spinal internal fixation surgery at a young age, the spine retains significant growth potential^13^. We measured relevant parameters of the vertebral body and spinal canal, utilizing adjacent vertebrae outside the fixed segment as a reference. The results indicated a slightly lower growth rate in the vertebral body’s width, length, and height within the fusion segment compared to the upper and lower adjacent vertebrae outside the fusion segment.

We calculated the Pv for estimation to gain further insights into vertebral body development. Our findings revealed a 72% increase in volume for UIV and a 67% increase for the LIV. In contrast, the adjacent vertebrae outside the fixed segment exhibited more substantial changes (92% for UNV and 74% for LNV). However, one-way ANOVA demonstrated a *p* value of 0.515 in the comparing Pv among different vertebrae, indicating that, despite variations in mean values, these differences lacked statistical significance. This suggests that the inhibitory effect of internal fixation on vertebral development is relatively minimal. The vertebral bodies within the fixed segment also exhibited growth during the follow-up period, possibly attributed to intact vertebral periosteum and at least one intact growth plate on either side^14^.

Furthermore, in assessing of spinal canal development, we measured the transverse and sagittal diameters of the spinal canal. The results showed that after a long-term follow-up, the changes of spinal canal diameters were much lower than the changes in the vertebral body. One-way ANOVA analysis suggested no statistically significant difference in the Pa among different vertebrae, which indicated that the impact of the internal fixation system on spinal canal development is minimal. Pedicle screws pass through the neural central cartilage (NCC) that connects the pedicle and vertebral body. Previous studies^13,15,16^ have shown that the NCC of the thoracic and lumbar spine in children starts to close at 4–5 years old, and there is not much change in the size of the spinal canal from then until adulthood. In a study by Olgun et al.^17^, no negative effect of internal fixation on spinal canal development was observed during a 2-year follow-up of children who had pedicle screws implanted before the age of 5. In our study, all surgically treated patients were aged 3 years or older. The measurements indicated a slight enlargement of the spinal canal during follow-up. However, there was no case of iatrogenic stenosis of the spinal canal or spinal cord compression resulting from the restriction of internal fixation. No neurological dysfunctions were observed in any of the patients after surgery or during the follow-up period.

### Limitations

This study has specific limitations. Some patients were too young before surgery to provide subjective assessments, such as satisfaction with appearance before the operation. Furthermore, the measurement of vertebral and spinal canal parameters for all patients relied on X-rays, introducing the potential for errors due to overlapping images, although these errors have been shown to be negligible in other studies^18^. More accurate CT scans are difficult to perform universally during long-term follow-up.

## Conclusion

In summary, the posterior approach hemivertebra resection and short-segment fusion surgery prove to be effective in correcting deformities. Early surgery can save fusion range without significant loss of correction during follow-up. Internal fixation at a young age shows no significant inhibitory effects on spinal development within the fusion segment. Hence, posterior hemivertebra resection and short-segment fusion surgery emerge as a safe and effective procedure.

## Methods

### Inclusion and exclusion criteria

Inclusion criteria: (1) Congenital scoliosis caused by a single thoracic or lumbar hemivertebra (fully or incompletely segmented) in children under 10 years of age, with indications for surgical treatment: segmental scoliosis angle greater than 25°, rapid progression and ineffective conservative treatment; (2) Surgical resection of the hemivertebra through a posterior approach and short-segment fusion fixation, with a fixation range of one vertebra above and below the hemivertebra; (3) Follow-up for more than 5 years; (4) Complete imaging data.

Exclusion criteria: (1) Surgery through anterior or anteroposterior approaches; (2) Unilateral pedicle screw internal fixation; (3) Presence of multiple vertebral bodies deformities or history of previous spinal surgery.

### Surgical methods

All surgical patients underwent surgery under neurologic monitoring. After endotracheal intubation under general anesthesia, the patient was placed in a prone position. The hemivertebra segment was exposed through a posterior midline incision, with full exposure of the posterior spinal structures, including the spinous process, lamina, and facet joints of the hemivertebra and the adjacent vertebrae above and below. Pedicle screws were placed in the adjacent vertebrae above and below the hemivertebra and temporarily fixed with a rod on the concave side. The lateral aspect of the hemivertebra was exposed along the base of the pedicle, and the hemivertebra and intervertebral disc were partially or completely excised based on the segmentation of the hemivertebra. Subsequently, a pre-contoured rod was placed on the convex side. Gradual compression was applied until the gap was successfully closed, addressing both the segmental scoliosis and kyphosis deformities for corrective purposes. C-arm radiography confirmed satisfactory correction, and spinal posterior column bone grafting was performed. The incision was closed layer by layer.

### Postoperative management

Strict bed rest is required for the first 2 weeks after surgery. The timing for the child to resume activities out of bed is determined based on the healing of the incision and postoperative imaging results. A plastic brace was provided for 3 months after the first ambulation, serving the purpose of protection and restriction of trunk movements to promote bone fusion. Imaging examinations are scheduled every 3 months during the first year postoperatively and every 6–12 months after the first year.

### Imaging data

For the assessment of orthopedic outcomes, we measure indicators including segmental scoliosis Cobb angle, cranial and caudal compensatory curves, coronal balance distance (CBD), segmental kyphosis (SK), thoracic kyphosis (TK), lumbar lordosis (LL), Sagittal vertical axis (SVA), and tilt angles of upper instrumented vertebra (UIV) and lower instrumented vertebra (LIV). Comparisons between different time points, including preoperative, postoperative and follow-up, were conducted to evaluate the orthopedic effects. The specific definitions and measurement methods of these indicators are provided in Table 6.

**Table 6:** Description of measurements.

	Measurements	Description
Corrective outcome measurements	Segmental scoliosis	The Cobb angle between the vertebrae above and below the hemivertebra in coronal plane
	Cranial compensatory curve	The Cobb angle of the compensatory curve above the segmental scoliosis in coronal plane
	Caudal compensatory curve	The Cobb angle of the compensatory curve below the segmental scoliosis in coronal plane
	Coronal balance distance (CBD)	The horizontal distance between C7 plumb line and the central sacral vertical line (CSVL)
	UIV tilt angle	The inclination angle of the upper endplate of the upper instrumented vertebra (UIV) to the horizontal
	LIV tilt angle	The inclination angle of the lower endplate of the lower instrumented vertebra (LIV) to the horizontal
	Thoracic kyphosis	The angle between the superior endplate of T4 and the inferior endplate of T12 in sagittal plane
	Lumbar lordosis	The angle between the superior endplate of L1 and the superior endplate of S1 in sagittal plane
	Segmental kyphosis	The angle between the superior endplate of the vertebra above the hemivertebra and the inferior endplate of the vertebra below the hemivertebra in sagittal plane
	Sagittal vertical axis (SVA)	Distance between the posterior superior sacral endplate and the vertical line from the center of C7 in sagittal plane
Spinal growth measurements	Vertebral body height (VBH)	The average sagittal diameter of the anterior edge (H1) and posterior edge (H2) of vertebral body, VBH = (H1 + H2)/2
	Vertebral body width (VBW)	The average width of the upper endplate (W1) and lower endplate (W2) of the vertebral body in the coronal plane, VBW = (W1 + W2)/2
	Vertebral body length (VBL)	The average distance between the anterior edge (L1) and posterior edge (L2) of the vertebral body in the sagittal plane, VBL = (L1 + L2)/2
	Spinal canal width (CLAT)	Interpedicular diameter of the spinal canal
	anterior–posterior diameter of the spinal canal (CAP)	The distance from the posterior edge of the vertebral body to the lamina in the sagittal plane

**Figure 2:**
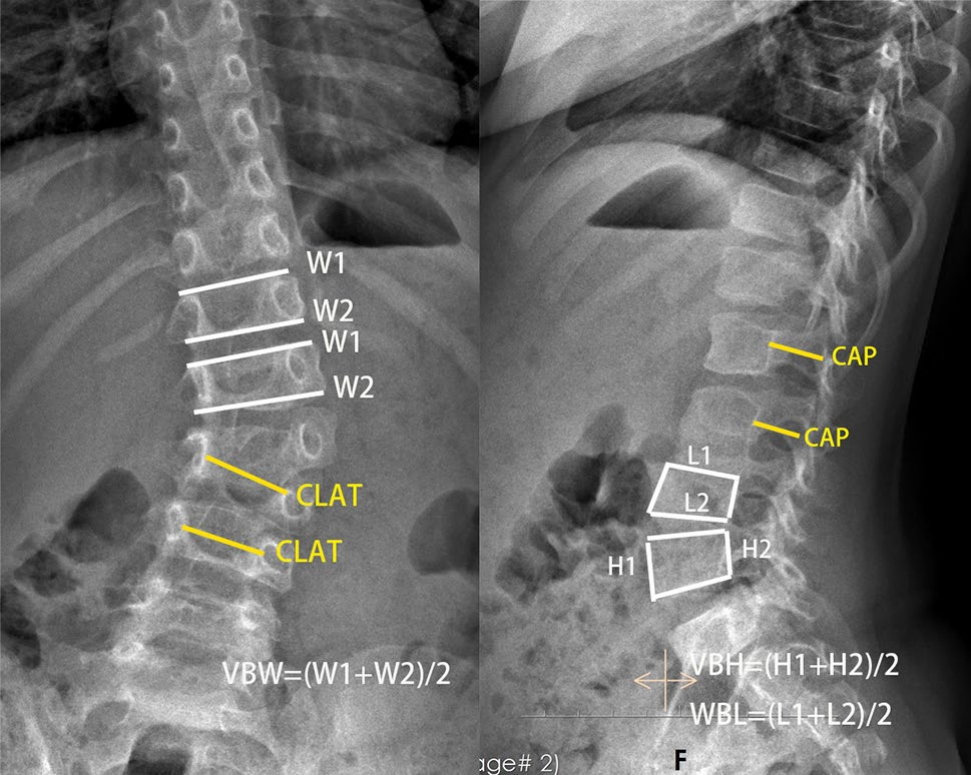
Illustration of measurements. (**a**) and (**b**) Illustration of vertebral body and spinal canal diameters measurements: W1: the width of the upper endplate of the vertebral body in the coronal plane; W2: the width of the lower endplate of the vertebral body in the coronal plane; H1: the height of the anterior edge of the vertebral body in sagittal plane; H2: the height of the posterior edge of the vertebral body in sagittal plane; L1: the length of the upper endplate of the vertebral body in sagittal plane; L2: the length of the lower endplate of the vertebral body in sagittal plane; CLAT: interpedicular diameter of the spinal canal ; CAP : The distance from the posterior edge of the vertebral body to the lamina in sagittal plane.

Vertebral development was assessed by measuring the diameters of the upper instrumented vertebra (UIV), lower instrumented vertebra (LIV), upper adjacent noninstrumented vertebra (UNV), and lower adjacent noninstrumented vertebra (LNV). Comparison of vertebral size parameters before and at the last follow-up was conducted to evaluate the development in different vertebrae. The diameters include vertebral body height (VBH), vertebral body width (VBW), vertebral body length (VBL), spinal canal width (CLAT) and anterior–posterior diameter of the spinal canal (CAP)^18^. The definitions and measurement methods are detailed in Table 6 and Fig. 2^19–21^.

In addition, the product of VBH, VBW and VBL was used to estimate the changes in vertebral volume before surgery and at the last follow-up, and the product of CLAT and CAP was used to estimate the changes in the vertebral canal area before and at the last follow-up^20^.

The ratio of vertebral body volume at the last follow-up to vertebral volume before surgery (Pv) was estimated as follows:

1. $${\text{Pv}} = \frac{{{\text{VBW}}_{{{\text{pre-op}}}} \times {\text{VBH}}_{{{\text{pre-op}}}} \times {\text{VBL}}_{{{\text{pre-op}}}} }}{{{\text{VBW}}_{{{\text{LF}}}} \times {\text{VBH}}_{{{\text{LF}}}} \times {\text{VBL}}_{{{\text{LF}}}} }}$$
  (Preop: preoperative; LF: last follow-up).

The ratio of the spinal canal area at the last follow-up to the preoperative spinal canal area (Pa) was estimated as follows:

2. $${\text{Pa}} = \frac{{{\text{CAP}}_{{{\text{pre-op}}}} \times {\text{CLAT}}_{{{\text{pre-op}}}} }}{{{\text{CAP}}_{{{\text{LF}}}} \times {\text{CLAT}}_{{{\text{LF}}}} }}$$
  (Preop: preoperative; LF: last follow-up).

### Statistical analysis

Data analysis was performed using the SPSS 22.0 software package (IBM Corporation, USA). Paired sample t tests were used to compare preoperative, postoperative, and last follow-up data for corrective surgery-related information (segmental scoliosis angle, compensatory curve angle, CBD, VSA, LK, TL, LL, UIV tilt, LIV tilt). Paired sample t tests were also used to compare vertebral body development-related information (VBH, VBW, VBL, CLAT, CAP) between preoperative and last follow-up data. One-way ANOVA was used to compare differences in estimated Pv and Pa values between different vertebrae. Differences were considered statistically significant at a level of a < 0.05.

### Ethical approval

We confirm that all methods were carried out in accordance with the Declaration of Helsinki and its later amendments, and all protocols were approved by the Ethics Committee of Xiangya Hospital of Central South University (ethics approval number: 201703359). We confirm that written informed consent was obtained from all subjects.

## Data Availability

Some or all data, or code that support the findings of this study are available from the corresponding author upon reasonable request.
